# Pseudotumeur cérébrale révélant une sarcoïdose

**DOI:** 10.11604/pamj.2017.28.113.10471

**Published:** 2017-10-06

**Authors:** Mounira El Euch, Madiha Mahfoudhi, Wafa Skouri, Fethi Ben Hamida, Fatima Jaziri, Khaoula Ben Abdelghani, Sami Turki, Taïeb Ben Abdallah

**Affiliations:** 1 Service de Médecine Interne «A» Hôpital Charles Nicolle, Tunis, Tunisie; 2 Laboratoire de Recherche des Maladies Rénales (LR00SP01), Hôpital Charles Nicolle, Tunis, Tunisie Faculté de Médecine de Tunis, Tunisie

**Keywords:** Hypertension intracrânienne, tuberculose, IRM, pseudotumeur cérébrale, sarcoïdose, Intracranial hypertension, tuberculosis, MRI, pseudotumor cerebri

## Abstract

La sarcoïdose est une granulomatose multi viscérale d'étiologie inconnue qui peut revêtir des tableaux cliniques et radiologiques diverses. Les localisations cérébrales bien que rares, peuvent se présenter sous forme pseudo-tumorale trompeuse. Nous rapportons l'observation d'un jeune adulte Tunisien hospitalisé pour hypertension intracrânienne en rapport avec une lésion pseudotumorale radiologique qui a révélé une sarcoïdose systémique.

## Abstract (en)

Sarcoidosis is a multivisceral granulomatosis of unknown aetiology which may have various clinical and radiological manifestations. Cerebral sarcoidosis, although rare, can appears as a misleading pseudotumor. We report the case of a young Tunisian adult hospitalized for intracranial hypertension associated with pseudotumoral lesion on radiology, which was revealed to be systemic sarcoidosis.

## Introduction

L'atteinte neurologique au cours de la sarcoïdose est rare et atteint 5 à 6% des patients [1]. Elle se manifeste souvent par une méningite granulomateuse [2]. Une lésion pseudo-tumorale est exceptionnelle et représente un défi diagnostique surtout en l'absence de signes systémiques [3].

## Patient et observation

**Figure 1:**
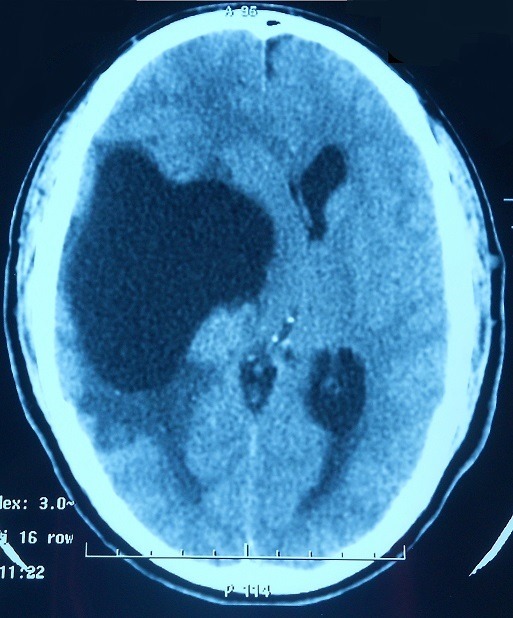
Cliché de TDM cérébrale montrant l'hypodensité temporale droite exerçant un effet de masse sur la ligne médiane avec œdème péri-lésionnel

**Figure 2:**
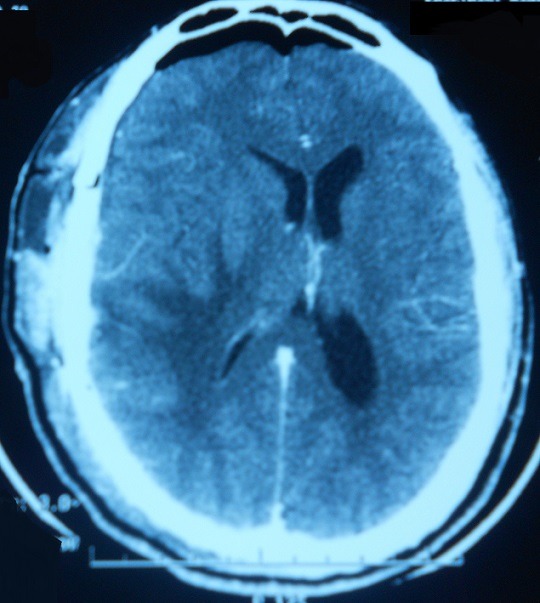
Cliché TDM cérébrale après corticothérapie qui montre la régression des lésions initiales

Il s'agit du patient MK âgé de 23 ans qui était admis initialement dans un tableau d'hypertension intracrânienne. L'examen clinique a objectivé des réflexes ostéotendineux vifs. L'examen ophtalmologique a montré un œdème papillaire. La biologie était sans anomalies. La TDM cérébrale avait montré une hypodensité temporale droite exerçant un effet de masse sur la ligne médiane avec œdème péri-lésionnel Figure 1. L'exérèse chirurgicale a montré un granulome tuberculoïde avec nécrose sans signes histologiques de malignité. Le diagnostic de tuberculose cérébrale a été retenu et le malade a été traité par des anti-tuberculeux pendant une année. L'évolution a été défavorable, marquée par l'apparition de crises convulsives et d'adénopathies cervicales et thoraco abdominales avec persistance de la lésion cérébrale à la TDM de contrôle. Le lavage broncho alvéolaire a révélé une alvéolite lymphocytaire avec rapport CD4/CD8=2.2. L'enzyme de conversion de l'angiotensine était augmentée. L'imagerie thoracique a révélé une pneumopathie interstitielle. Une nouvelle biopsie cervicale a conclu à un granulome sans nécrose caséeuse. Le diagnostic de neurosarcoïdose a été retenu. Il a été traité par prednisone à la dose de 1 mg/kg/j et méthotrexate à la dose de 15 mg/semaine avec évolution favorable cliniquement et radiologiquement Figure 2 selon un recul de 4 ans de suivi.

## Discussion

La sarcoïdose est une maladie multisystémique d'étiologie inconnue, caractérisée par la présence de granulomes non caséeux dans différents organes [4]. Contrairement à l'atteinte respiratoire qui est la plus fréquente, le système nerveux central est rarement touché. Cette atteinte peut revêtir plusieurs tableaux cliniques. La forma pseudo tumorale est exceptionnelle et n'a été rapportée que dans quelques cas [5-9] Il est important de reconnaître les diagnostics différentiels des pseudotumeurs cérébrales tels que les méningiomes de la base du crâne et les tumeurs sellaires parce que la résection chirurgicale agressive n'est pas indiquée [4]. Pour notre cas, la tuberculose cérébrale était évoquée en premier lieu et l'absence de réponse aux anti-tuberculeux en plus des signes systémiques a permis de redresser le diagnostic qui reste litigieux. La confirmation histologique n'est pas de ce fait indispensable pour confirmer le diagnostic de la neurosarcoïdose. La corticothérapie prolongée aboutit souvent à une réponse clinique plus ou moins complète, attestée par la régression des lésions actives identifiées sur l'IRM.

## Conclusion

La sarcoïdose peut exceptionnellement simuler un syndrome tumoral dans sa forme multi nodulaire. La preuve histologique est nécessaire afin d'éliminer d'autres étiologies notamment tumorales. Il existe fréquemment une discordance entre l'atteinte radiologique et la présentation clinique.

## Conflits d’intérêts

Les auteurs ne déclarent aucun conflit d'intérêt.
